# Alliance for Clinical Trials in Oncology (Alliance) trial A022101/NRG-GI009: A pragmatic randomized phase III trial evaluating total ablative therapy for patients with limited metastatic colorectal cancer: evaluating radiation, ablation, and surgery (ERASur)

**DOI:** 10.21203/rs.3.rs-3773522/v1

**Published:** 2023-12-23

**Authors:** Kathryn E Hitchcock, Eric D. Miller, Qian Shi, Jesse G. Dixon, Sepideh Gholami, Sarah B. White, Christina Wu, Christopher C. Goulet, Manju George, Kyung-Wook Jee, Chadwick L. Wright, Rona Yaeger, Ardaman Shergill, Theodore S. Hong, Thomas J. George, Eileen M. O’Reilly, Jeffrey A. Meyerhardt, Paul B. Romesser

**Affiliations:** University of Florida; The Ohio State University; Alliance for Clinical Trials in Oncology; Alliance for Clinical Trials in Oncology; Northwell Health; Medical College of Wisconsin; Mayo Clinic Arizona; Billings Clinic; Colontown/ Platown Development Foundation; Massachusetts General Hospital; University of Cincinnati; Memorial Sloan Kettering Cancer Center; Alliance for Clinical Trials in Oncology; Massachusetts General Hospital; University of Florida; Memorial Sloan Kettering Cancer Center; Dana-Farber Cancer Institute; Memorial Sloan Kettering Cancer Center

**Keywords:** colorectal cancer, oligometastatic disease, radiation, stereotactic, chemotherapy, microwave ablation, clinical trial

## Abstract

**Background:**

For patients with liver-confined metastatic colorectal cancer (mCRC), local therapy of isolated metastases has been associated with long-term progression-free and overall survival (OS). However, for patients with more advanced mCRC, including those with extrahepatic disease, the efficacy of local therapy is less clear although increasingly being used in clinical practice. Prospective studies to clarify the role of metastatic-directed therapies in patients with mCRC are needed.

**Methods:**

The Evaluating Radiation, Ablation, and Surgery (ERASur) A022101/NRG-GI009 trial is a randomized, National Cancer Institute-sponsored phase III study evaluating if the addition of metastatic-directed therapy to standard of care systemic therapy improves OS in patients with newly diagnosed limited mCRC. Eligible patients require a pathologic diagnosis of CRC, have *BRAF* wild-type and microsatellite stable disease, and have 4 or fewer sites of metastatic disease identified on baseline imaging. Liver-only metastatic disease is not permitted. All metastatic lesions must be amenable to total ablative therapy (TAT), which includes surgical resection, microwave ablation, and/or stereotactic ablative body radiotherapy (SABR) with SABR required for at least one lesion. Patients without overt disease progression after 16–26 weeks of first-line systemic therapy will be randomized 1:1 to continuation of systemic therapy with or without TAT. The trial activated through the Cancer Trials Support Unit on January 10, 2023. The primary endpoint is OS. Secondary endpoints include event-free survival, adverse events profile, and time to local recurrence with exploratory biomarker analyses. This study requires a total of 346 evaluable patients to provide 80% power with a one-sided alpha of 0.05 to detect an improvement in OS from a median of 26 months in the control arm to 37 months in the experimental arm with a hazard ratio of 0.7. The trial uses a group sequential design with two interim analyses for futility.

**Discussion:**

The ERASur trial employs a pragmatic interventional design to test the efficacy and safety of adding multimodality TAT to standard of care systemic therapy in patients with limited mCRC.

## Background

For patients with metastatic colorectal cancer (mCRC), surgical resection, when possible, has been associated with long-term progression-free survival (PFS) and overall survival (OS). Several large retrospective series have demonstrated 5-year OS rates of 40–70% in patients with isolated liver metastasis following liver metastasectomy [1–5]. Indeed, improvements in OS in patients with newly diagnosed mCRC over the last several decades have been attributed, in part, to an increase in hepatic resection [6]. For patients with limited extrahepatic disease, complete surgical resection has also been associated with prolonged PFS and OS, although the data are more limited [7–9]. While prospective evidence is lacking, these retrospective studies have demonstrated excellent long-term survival for patients with resectable mCRC and have defined the current standard of care (SOC).

In patients with multiorgan oligo-mCRC (e.g., low burden but liver inoperable disease or minimal extrahepatic and/ or extra-thoracic disease), it is less clear whether local ablative therapies, including thermal ablation and stereotactic ablative radiation therapy (SABR), can provide clinical benefit such as durable control of disease or improve survival. Limited prospective data exist on the benefit of thermal ablation to all areas of inoperable hepatic disease. The CLOCC phase II randomized trial (EORTC-40004) demonstrated that the addition of radiofrequency ablation to systemic therapy improved OS in patients with mCRC with inoperable liver disease (hazard ratio [HR] 0.58, 95% confidence interval [CI] 0.38–0.88, *p* = 0.01) [10, 11]. Multiple mature retrospective series have also reported high rates of local control and favorable long-term survival following the use of thermal ablation for CRC liver metastases [12–15].

SABR appears to be a safe and effective way to treat multiple metastatic sites in the lung, abdomen/pelvis, bone, and spine [16]. The use of SABR for the treatment of CRC lung and liver metastases has demonstrated local control rates of 80–90% with minimal toxicity [17, 18]. Additionally, there is emerging evidence that SABR to all sites of radiographic disease may improve PFS and OS [17, 18]. SABR-COMET was a randomized, phase II trial that demonstrated improved OS with SABR compared to the standard of care arm (median OS 41 vs. 28 months, HR 0.57, 95% CI 0.30–1.10, *p* = 0.090), although having 4.5% grade 5 treatment-related adverse events in the SABR arm. While SABR-COMET was designed as a tumor-agnostic trial in 99 patients with up to 5 metastatic lesions, 27% (n = 9) of the patients in the control group and 14% (n = 9) in the SABR group had a CRC primary [19, 20]. In addition to SABR-COMET, multiple other trials have shown the benefit of SABR in the oligometastatic setting including non-small cell lung cancer, prostate, and renal cell carcinoma [21–25]. These studies demonstrate that SABR is a safe and highly effective locoregional therapy that improves oncologic outcomes in a variety of disease settings, including in some settings where an oligometastatic paradigm has not been well established, in contrast to CRC. In fact, to date there are no completed randomized clinical trials investigating the benefit of SABR in patients with mCRC.

High quality data on the utilization of multimodality metastatic-directed therapy, including the combination of surgical resection, thermal ablation, and SABR, for patients with mCRC is limited despite increased use in clinical practice. A recent prospective Finnish interventional study (RAXO study) highlights the potential for multimodality directed therapy in patients with metastatic CRC [26]. The 5-year OS for patients treated with systemic therapy alone was 6% compared to 40% for patients treated with local ablative therapies and/ or surgical debulking (i.e., R2 resection) [26]. A 5-year survival of 40% with multimodality metastatic-directed therapy is quite notable, as for context the 5-year survival for patients who underwent metastasectomy (R0/R1 resection) was in comparison 66%. These data suggest that local metastatic-directed therapy with SABR, thermal ablation, and surgery may significantly enhance cancer control and enhance overall survival in patients with mCRC rather than continuing with systemic therapy alone.

ERASur was jointly developed by the National Cancer Institute (NCI)’s Alliance for Clinical Trials in Oncology and NRG Oncology to evaluate multimodality metastases-directed therapy in patients with mCRC. Despite the long history of treating oligometastatic CRC, questions remain regarding the benefit of extending local metastatic-directed therapies to patients with more extensive metastatic disease including in patients with extrahepatic disease. The ERASur trial seeks to fill this gap by testing if total ablative therapy (TAT) to all sites of metastatic disease improves survival using a pragmatic design that integrates the current spectrum of multimodality local therapies. If the addition of TAT to SOC systemic therapy improves OS, it will be established as a new standard of care for patients with limited mCRC. If TAT is associated with increased toxicity without improving OS, then future treatment paradigms can avoid unnecessary toxicity associated with TAT.

## Methods/Design

### Study Objectives

The primary objective of ERASur is to compare the outcome of using TAT in addition to SOC systemic therapy versus SOC systemic therapy alone in terms of OS, measured from the time of randomization, in patients with newly diagnosed limited mCRC. The secondary objectives include evaluating event-free survival, adverse events, and time to local recurrence for patients treated with TAT, defined as the time from the end of TAT to the date of first documented recurrence at any disease site treated with TAT.

### Study Setting

ERASur is co-led by the Alliance for Clinical Trials in Oncology and NRG Oncology through the NCI National Clinical Trials Network (NCTN) and supported by the Southwest Oncology Group (SWOG) and the Eastern Cooperative Oncology Group-American College of Radiology Imaging Network (ECOG-ACRIN) Cancer Research Group. Patients will be accrued from member institutions of these NCTN cooperative groups which includes community and academic sites. NCI Central Institutional Review Board (CIRB) approved the study, with participating institutions relying on the CIRB. All patients must provide written informed consent.

### Study Design

**Figure 1:**
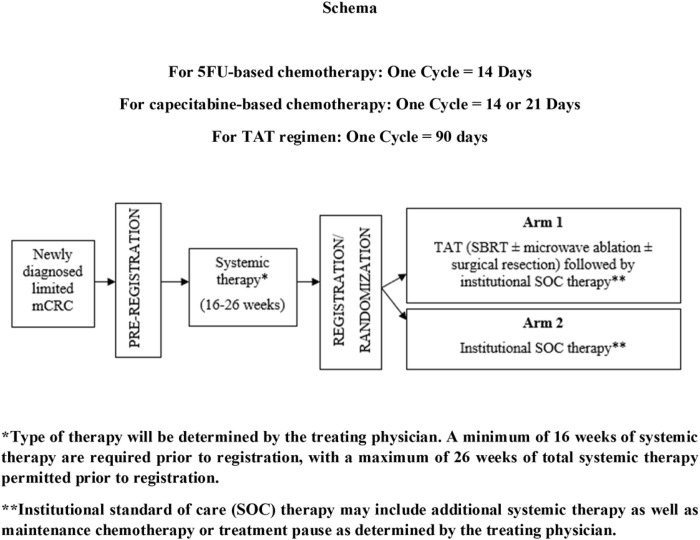
ERASur trial schema.

ERASur is a two-arm, multi-institutional, randomized phase III study investigating the effect of the addition of TAT to SOC systemic therapy in patients with limited mCRC. The study schema is illustrated in Figure 1.

### Patient Selection and Eligibility Criteria

Patients 18 years of age or older with histologically confirmed mCRC with 4 or fewer sites of metastatic disease are eligible. Metastatic sites must be radiographically evident, but pathologic confirmation is not required. Single sites include: each hemi-liver (right and left), each lobe of the lungs, each adrenal gland, lymph nodes amenable to a single resection or treatment in a single SABR field, and bone metastases amenable to treatment in a single SABR field. Patients with liver-only metastatic disease are not eligible, nor are patients whose tumors are known to have *BRAF V600E* mutations or microsatellite unstable. Metastatic lesions must be amenable to any combination of surgical resection, microwave ablation (MWA), and/or SABR. SABR is required to at least one site. Detailed eligibility criteria are shown in Table 1. Patients will have the option of pre-registering for the study within 16 weeks of starting first-line SOC systemic therapy with regimens including 5-fluorouracil, leucovorin, and oxaliplatin (mFOLFOX6), capecitabine and oxaliplatin (CAPOX), 5-fluorouracil, leucovorin, and irinotecan (FOLFIRI), and 5-fluorouracil, leucovorin, oxaliplatin, and irinotecan (mFOLFOXIRI) with or without anti-VEGF or EGFR therapies. For registration, a minimum of 16 weeks and a maximum of 26 weeks of first-line systemic therapy is required. Patients with overt disease progression after 16–26 weeks of first-line systemic therapy are not eligible for the study and if pre-registered will be removed. The study calendar is shown in Table 2.

### Treatment Plan

Upon registration, which occurs after completing a minimum of 16 weeks or a maximum of 26 weeks of first-line systemic therapy, patients will be randomized to one of two treatment arms. Patients in arm 1 will undergo TAT followed by SOC chemotherapy per institutional practice. For patients in arm 1, the overall treatment plan will be discussed in a multidisciplinary setting, and the patient will be evaluated by physicians from all planned treatment modalities as early as possible for treatment planning. TAT will consist of surgical resection, MWA, and/or SABR to all sites of disease and must be completed within 90 days from randomization. At least one measurable site of metastatic disease needs to be present after completion of induction systemic therapy for treatment, and patients with a complete response to systemic therapy at time of randomization will be removed from the trial. At least one metastatic site must be treated with SABR. The remaining sites can be treated by either SABR with or without surgery and/or MWA. For treatment with SABR, the goal is to deliver a radiation dose that maximizes local control at the treatment site within the confines of anatomic and normal tissue constraints. However, sites must be credentialed for the treatment modality that they intend to use on all patients. All radiation therapy plans will be reviewed in real-time for quality assurance.

Resection of each planned metastatic lesion will be approached with the intent of an R0 resection. If surgical resection of a given metastasis is incomplete with gross or microscopic residual margins, the treatment team should strongly consider using an alternative ablative treatment modality such as MWA or SABR to any residual gross or microscopic disease. When addressing liver metastases, non-anatomic resection will be considered when feasible, and MWA will be considered to allow for a parenchymal-sparing approach for deep lesions less than 3 cm in size. For all patients who undergo surgery during protocol treatment, the preoperative imaging, operative note, surgical pathology report, and adverse events with 30 days of surgery will be reviewed by the study team for quality assurance.

MWA can be delivered either intra-operatively or using a percutaneous approach. Multiple electrodes and overlapping ablations will be permitted to ensure adequate coverage of the target. A minimum margin of mm will be required for lesions treated with MWA on this study. Initial assessment of the ablation zone will be verified immediately intra-procedurally using ultrasound, computed tomography (CT), or magnetic resonance imaging (MRI). If a margin of <5.0 mm is observed at initial assessment, additional ablation will be attempted to extend the ablation zone, expand the area of insufficient coverage and provide for at least 5.0 mm minimal margin around the target tumor. If at the first imaging timepoint the tumor is deemed to be incompletely covered, the tumor can undergo repeat treatment without penalization. As quality assurance, the study team will review pre-treatment imaging, the procedure notes, and adverse events within 30 days of treatment associated with MWA. Imaging from the first assessment timepoint at 14–18 weeks post-randomization will also be reviewed to ensure completion of planned ablation.

Lesions that are too small to be treated with any of the modalities included in TAT will be monitored and treated if they progress to a size that is amenable to treatment, and they will not be considered as Response Evaluation Criteria in Solid Tumors (RECIST) progression. Following completion of TAT, the treating healthcare team will consider re-starting systemic therapy within 2 weeks if no surgery is performed or within 4 weeks if surgery is included as part of TAT. Use of maintenance systemic therapy or systemic therapy breaks is permitted at the discretion of the treatment team. Patients randomized to arm 1 with the primary tumor intact will have the primary tumor removed within 6 months of randomization. Resection of the primary tumor may be performed at the same time as metastasectomy or may be staged per discretion of the healthcare team. For patients with primary rectal cancers, the use of pre-operative radiation or chemoradiation will be left to the discretion of the healthcare team.

Patients randomized to arm 2 will continue with systemic therapy with use of maintenance chemotherapy per institutional practice. Local metastatic-directed therapy will not be permitted except for palliation as per institutional standard practices. Palliative radiation therapy will be permitted for lesions causing symptoms that are not controlled by medical therapy with acceptable regimens including 30 Gy in 10 fractions, 24 Gy in 6 fractions, 20 Gy in 5 fractions, 8 Gy in 1 fraction, or an equivalent regimen. Systemic therapy breaks are permitted at any time at the discretion of the treatment team.

### Assessment and Follow-up

Radiologic response will be evaluated using the RECIST version 1.1 guidelines [27]. A local recurrence will be defined differently based on the modality of treatment. For patients treated with SABR, a recurrence will be deemed local if located in or directly adjacent to the planning target volume. For a site treated using MWA, a recurrence will be deemed local if it is within 1 cm of the treatment site. For patients who undergo surgery, a recurrence will be considered local if it is located at the margin of resection.

Adverse events (AEs) will be graded using the Common Terminology Criteria for Adverse Events (CTCAE) Version 5.0. Solicited AEs will be collected at baseline prior to treatment until off treatment. Routine AEs will be collected starting after registration until the end of survival follow-up. The first treatment response assessment timepoint will be at 14–18 weeks post-randomization, and then every 3 months until disease progression or at the start of off-protocol anticancer therapy. Off-protocol anticancer therapies consists of any investigational agent, systemic therapy regimen(s) not included in the protocol; for patients randomized to arm 2, this includes any local metastatic-directed therapy other than therapy delivered with palliative intent. All visits will include a history and physical examination, laboratory studies, AE assessment, and imaging with CT of the chest along with CT or MRI of the abdomen and pelvis or, alternatively, a positron emission tomography/computed tomography (PET/CT). All patients, irrespective of whether continuing on study, or who are receiving off protocol therapy, will be followed for OS (except patients who withdraw consent).

### Correlative Studies

Patients may elect to consent to collection of blood and archival formalin-fixed paraffin-embedded tissue for future genomic analyses. Three 10 mL blood samples will be collected at several time points including within 14 days of pre-registration for those who enroll prior to initiating systemic therapy, at randomization, at 4 months, 8 months, and 1 year after randomization, and at disease progression.

### Statistics

#### Sample Size

Per study design, a total of 346 patients (173 per arm) are needed to evaluate the primary endpoint. An additional 18 patients (5% inflation) will be accrued to allow for withdrawal after randomization and major violations. Thus, the total planned target accrual will be 364 patients. Approximately 405 patients will be pre-registered to reach this target accrual, allowing for a 10% drop out during the initial 16 to 26 weeks of SOC systemic therapy due to complete response status, progressive disease, unacceptable toxicity, patient withdrawing consent, treating physician’s decision, etc. With an anticipated accrual of 6.5 patients per month, we estimate the accrual period to be 4.7 years.

#### Power Analysis

Eligible patients will be stratified by the number of metastatic organ sites (1–2 vs. 3–4), timing of metastatic disease diagnosis (synchronous metastatic disease vs. metachronous metastatic disease diagnosed ≥12 months following completion of definitive treatment for initial diagnosis), and presence of at least one metastatic site outside the liver and lungs (yes vs. no). Participants will be assigned to one of two treatment arms in a 1:1 ratio, using a dynamic allocation algorithm [28]. This study will utilize a group sequential design with two interim analyses for futility after observing 25% (52 events) and 50% (104 events) of events, adopting the Rho family (Rho=1.5) beta spending function for controlling the type II error rate. Based on historical data, the median OS is assumed to be 26 months (following 16–26 weeks of initial SOC systemic therapy) for newly diagnosed mCRC patients treated with SOC systemic therapy. We assume an accrual rate of 6.5 patients per month, minimum follow-up on all patients of 60 months, exponential survival, and a one-sided log-rank test for superiority conducted at a one-sided significance level of 0.05. Based on these assumptions, a total number of 208 events will provide 80% power to detect an HR of 0.7 at a one-sided significance level of 0.05 requiring randomization of at least 346 evaluable patients (173 per arm).

## Discussion

ERASur is a multicenter randomized phase III clinical trial currently accruing through the U.S. NCI NCTN, which is designed to evaluate the benefit of adding metastatic-directed therapy to SOC systemic therapy in patients with limited mCRC. As imaging and treatment technology and techniques improve, the ability to detect and safely treat metastatic disease with local therapy has improved. However, carefully designed prospective randomized trials are necessitated to fully inform the value of this strategy with regard to efficacy, safety, costs and other consideration. The results of ERASur will help to define the clinical utility of TAT in patients with limited mCRC with extrahepatic disease. The trial activated in January 2023 through the NCI Cancer Trials Support Unit and is currently enrolling.

The conceptualization and design of ERASur was co-led by the Alliance for Clinical Trials in Oncology and NRG Oncology. The study incorporated input from a multidisciplinary team, comprised of experts in surgical, medical, radiation, interventional radiology, imaging and other disciplines, including patient advocacy, which was particularly important given the varied therapeutic modalities under investigation. The final study design was forged with critical input from the NCI Colon Task Force, alongside the guidance of the NCTN including the NCI Gastrointestinal steering committee and Cancer Therapy Evaluation Program. Patient advocates provided input early in the trial design in collaboration with COLONTOWN, a large online community of patients who have had or currently have CRC. Patient engagement and input was sought through multiple online polls run independently by COLONTOWN leadership in order to assist with key study design questions and to gauge patient interest for the trial within the COLONTOWN community [29]. The COLONTOWN community showed exceptionally strong support for ERASur with 90% of patients (N = 127) stating an interest in participating on the trial if they were eligible.

While inception of the trial required multidisciplinary input, successful completion of the trial will also require a concerted effort of the treatment teams at participating sites. For patients randomized to the TAT experimental arm, the selection and sequencing of metastatic-directed therapy will largely be left to the individual healthcare teams within the protocol’s guidance, including use of SABR for at least one site and surgery reserved for lung, liver, and portocaval lymph nodes. This design is by intent, both to maintain the pragmatic nature of this study and to reflect ‘real world’ clinical practice. Rigorous quality assurance mechanisms are in place in addition to two interim analyses to ensure that patients are treated safely with sufficient thresholds for stopping the study for futility.

ERASur is a study that could only be designed and conducted in a cooperative group setting with federal support. Specifically, the primary study hypothesis does not involve an investigational therapeutic or a new device, lending itself relatively unsuitable to pharmaceutical or device manufacture sponsorship. This trial has the potential to significantly impact practice with a positive result providing the much-needed high level evidence to support the practice of integration of TAT in mCRC, and a negative or neutral outcome of this strategy suggesting that SOC systemic therapy is a preferred approach for most patients.

## Data Availability

The datasets generated and analyzed during the current study will be available in Medidata/RAVE repository and will be available on reasonable request and pursuant to Alliance for Clinical Trials in Oncology guidelines. Please contact Dr. Hitchcock (hitcka@shands.ufl.edu), Dr. Miller (eric.miller@osumc.edu), or Dr. Romesser (romessep@mskcc.org) to request the data from this study.

## Abbreviations

AEs: adverse events
CAPOX: capecitabine and oxaliplatin
CIRB: Central Institutional Review Board
CRC: colorectal cancer
CT: computed tomography
CTCAE: Common Terminology Criteria for Adverse Events
ECOG-ACRIN: Eastern Cooperative Oncology Group-American College of Radiology Imaging Network
ERASur: Evaluating Radiation, Ablation, and Surgery
FOLFIRI: 5-fluorouracil, leucovorin, and irinotecan
HAIP: hepatic artery infusion pump
mCRC: metastatic colorectal cancer
mFOLFOX6: 5-fluorouracil, leucovorin, and oxaliplatin
mFOLFOXIRI: fluorouracil, leucovorin, oxaliplatin, and irinotecan
MRI: magnetic resonance imaging
MSI: microsatellite instable
MWA: microwave ablation
NCI: National Cancer Institute
NCTN: National Clinical Trials Network
OS: overall survival
PET/CT: positron emission tomography/computed tomography
PFS: progression-free survival
RECIST: Response Evaluation Criteria in Solid Tumors
SABR: stereotactic ablative body radiotherapy
SOC: standard of care
SWOG: Southwest Oncology Group
TAT: total ablative therapy
ULN: upper limit of normal
